# Book Review

**Published:** 1950-07

**Authors:** 


### Reviews of Books

**The Rhesus factor.** By G. FULTON ROBERTS, M.B. Second Edition. Pp. vii, 64. London: William Heinemann Medical Books Ltd. 1949. 3s. 6d.—The new edition of this book has been revised and enlarged. It is primarily a book for the general medical public: in it the essential facts are presented lucidly, without fuss and without that dogmatism under which complex subjects are so often made to masquerade as simple. Of the classifications Murray's has been wisely omitted; there is confusion enough between Weiner's and that of Fisher now generally used in this country. Dr. Roberts has not sufficiently stressed the significance of this : on page 36 he states: " It can be seen that Rhesus Positivity is synonymous with D Positivity . . . the term Rhesus positive means that the D antigen is present in the Genotype." This is not true and that Dr. Roberts is aware of it is evident later in the chapter. The confusion arises in attempting to equate the terms, " Rhesus positive and Rhesus negative " with " D positive and D negative ", a procedure not without danger. On pages 6 and 46 *rh* is referred to as a Mendelian recessive. This is surely incorrect. In a later edition it might be wiser for Dr. Roberts to abandon the terms Rhesus positive and Rhesus negative, and confine himself to Fisher's classification. On the whole, however, this is a valuable and well constructed little book.

**Diabetes.** By JOSEPH H. BARACH, M.D., F.A.C.P. Pp. xviii, 326. Illustrated. New York: Oxford University Press. 1949. 50s.—This new book on *Diabetes and Its Treatment* by Dr. Joseph H. Barach, offers to the general medical man a clinical approach to the understanding of diabetes. The outline of the aetiological factors involved in diabetes is clear and well illustrated and modern ideas concerning the disease are briefly described. Much use is rightly made of the author's extensive experience, though full note has not always been made of the work by other schools. In the statistical tables no attention is given to the possibility that diabetes mellitus may only represent a syndrome and may consist of one or more disease entities. In treatment the author evidently adheres to the " chemical " school, being satisfied with nothing less than the maintenance of a blood glucose level of less than 175 mg. per 100 mil. Insufficient stress is placed on the beneficial effects of a reducing diet in obese diabetics. A feature of the book is a section of detailed diets: there are 126 such diets for men, and 126 for women, forty-one for children, eight luncheon menus, and fifteen emergency diets; these are evidently duplicated in a companion book for patients. Whether such a detailed use of diet in the treatment of diabetes, especially for those receiving insulin, is either justifiable or useful is an open question. Also, of course, the diets given are not obtainable at the present time in this country. The book will not be of great use to those who are experienced in treating diabetics, but for those who wish to have an outline of modern theories on diabetes it should have some appeal. There is, however, little discussion of the practical points involved in the stabilization of the really difficult cases.

**William Withering of Birmingham.** By T. WHITMORE PECK, M.P.S., and K DOUGLAS WILKINSON, O.B.E., M.D., F.R.C.P. Pp. 240. Illustrated. Bristol: John Wright and Sons Ltd. 1950. Price 21s.—It was no doubt fortunate that Dr. Withering, in 1775, was a botanist of renown when he was presented with the problem of what was the substance in an old woman's cure for the dropsy, as then in use in Shropshire. His arresting reply was that " it was not very difficult for one conversant with these subjects to perceive that the active herb could be no other than the Foxglove". This was the beginning of all that has gradually led up to the modern pharmacology and uses of this indispensable substance. Withering deserves every credit for his carefully recorded and meticulous clinical observations on the action of digitalis; not least upon the " lady with the weak and intermittent pulse, breath very short and laborious, and her stomach, legs and thighs greatly swollen . . . " who after five draughts of Decoct-Digitalis " made upwards of eight quarts of water in the first twenty-four hours, and was thus snatched from impending destruction ". There is something of the Hippocratic tradition in this. In this beautifully produced book " William Withering of Birmingham ", Peck and Wilkinson have gathered practically all that is known about this distinguished physician and the story of an arduous and somewhat heroic life incidentally records a good deal about the way of life of the consultant in the latter half of the eighteenth century. The book should be of absorbing interest to every doctor and probably many will afterwards be encouraged to turn to and read Withering's original " Account of the Foxglove ".

**Diagnosis of Hysteria.** By D. W. ABSE, M.D., B.Sc., D.P.M. Pp. xii, 112. Illustrated. Bristol: John Wright and Sons Ltd. 1950. Price 8s. 6d.—This book describes Hysteria from the Freudian angle. The illustrations are well chosen from the author's service cases in Great Britain and India. It is clearly written and covers the subject very widely. But the author does not, himself, make any new contribution to the subject. It is doubtful whether the diagnosis of Hysteria justifies a book in itself. The author's main points, expressed in some thirty pages, would provide a most useful chapter in any textbook on psychoneurosis.

**Asthma.** By CLEMENT FRANCIS, M.A., M.B., B.CH. Pp. 49. Illustrated. London: Wm. Heinemann Medical Books Ltd. 1950. Price 5s.—The author of this funny little pamphlet proclaims himself "Surgeon i/c

Allergy Clinic "! The work itself is an account of what can be done with the nasal cautery: for example, lasting reduction of systolic pressure from 175 to 135!! As the Iron Duke said, " Sir: If you can believe that . . . "

**Operative Surgery.** Edited by Alexander Miles, M.D., LL.D., F.R.C.S. and Sir James Learmonth, K.C.V.O., C.B.E., Ch.M., F.R.C.S. Third Edition. Pp. x, 559. Illustrated. London: Oxford University Press (Geoffrey Cumberlege). 1950. Price 30s.—This is a third edition of the book on operative surgery which replaced the well-known " Thompson and Miles ", of long ago. It is by twenty-four contributors, all of whom are or have been connected with Edinburgh. It is addressed to students, house surgeons and presumably registrars, and gives all the information they are likely to find useful in the compass of 550 pages, well turned out and illustrated. We note that the sections on amputations, and on abdominal surgery in general, have been rewritten. The growing custom of getting the abdominal patient up in a day or two is not favoured. The book will be useful to experienced surgeons sometimes, but unusual or recent operations are unevenly dealt with. Many are included, but others (for instance operations on the central nervous system, etc.) are omitted. The author exposes the splenic vessels so that they can be digitally controlled before embarking on a splenectomy: and recommends oesophago-gastrostomy for cardiospasm.

**The Tuberculous Process.** By A. Leitch, M.B., Ch.B. Pp. viii, 175. Illustrated. Bristol: John Wright & Sons Ltd. 1949. Price 12s. 6d.—This book appears to have little practical value.

